# Readiness to implement on-site molecular testing for tuberculosis in community health centers in Uganda

**DOI:** 10.1186/s43058-022-00260-y

**Published:** 2022-02-02

**Authors:** Talemwa Nalugwa, Margaret Handley, Priya Shete, Christopher Ojok, Mariam Nantale, Tania Reza, Achilles Katamba, Adithya Cattamanchi, Sara Ackerman

**Affiliations:** 1 Department of Medicine, School of Medicine, Makerere University College of Health Sciences, Kampala, Uganda; 2 Uganda Tuberculosis Implementation Research Consortium, Kampala, Uganda; 3 Department of Epidemiology and Biostatistics, University of California San Francisco, San Francisco, CA USA; 4 Center for Vulnerable Populations, University of California San Francisco, San Francisco, San Francisco, CA USA; 5 Center for Tuberculosis, University of California San Francisco, San Francisco, CA USA; 6 Division of Pulmonary and Critical Care Medicine, San Francisco General Hospital, University of California San Francisco, San Francisco, CA USA; 7 Department of Social and Behavioral Sciences, University of California San Francisco, San Francisco, CA USA

**Keywords:** Tuberculosis, Onsite molecular testing, GeneXpert, Xpert MTB/RIF, Health systems, Uganda, Readiness

## Abstract

**Background:**

Newer molecular testing platforms are now available for deployment at lower-level community health centers. There are limited data on facility- and health worker-level factors that would promote successful adoption of such platforms for rapid tuberculosis (TB) testing and treatment initiation. Our study aimed to assess readiness to implement onsite molecular testing at community health centers in Uganda, a high TB burden country in sub-Saharan Africa.

**Methods:**

To understand implementation readiness, we conducted a qualitative assessment guided by the Consolidated Framework for Implementation Research (CFIR) at 6 community health centers in central and eastern Uganda between February and April 2018. We conducted 23 in-depth, semi-structured interviews with health workers involved in TB care at each health center to assess TB-related work practices and readiness to adopt onsite molecular testing using the GeneXpert Edge platform. Interviews were transcribed verbatim and coded for thematic analysis.

**Results:**

Participants (*N*=23) included 6 nurses/nursing assistants, 6 clinicians, 6 laboratory directors/technicians, 1 medical officer, 2 health center directors, and 2 other health workers involved in TB care. Health workers described general enthusiasm that on-site molecular testing could lead to greater efficiencies in TB diagnosis and treatment, including faster turn- around time for TB test results, lack of need for trained laboratory technicians to interpret results, and reduced need to transport sputum specimens to higher level facilities. However, health workers also expressed concerns about implementation feasibility. These included uncertainty about TB infection risk, safety risks from disposal of hazardous waste, a lack of local capacity to provide timely troubleshooting and maintenance services, and concerns about the security of GeneXpert devices and accessories. Health workers also expressed the need for backup batteries to support testing or charging when wall power is unstable.

**Conclusion:**

Our study generated a nuanced understanding of modifiable contextual barriers and led to direct revisions of implementation strategies for onsite molecular testing. The findings highlight that novel diagnostics should be implemented along with health system co-interventions that address contextual barriers to their effective uptake. Pre-implementation assessment of stakeholder perspectives, collaborative work processes, and institutional contexts is essential when introducing innovative technology in complex health care settings.

Contributions to the literature

- There are few studies reporting on readiness to implement health interventions from low-income countries. We demonstrate the application of CFIR—a widely used implementation science framework—to identify potential modifiable barriers to implementation of onsite molecular testing for TB at community health centers in a high TB burden setting.
- Even though newer molecular testing platforms have been designed to overcome infrastructure limitations, additional factors may influence their uptake and effective implementation.
- Implementation strategies for onsite molecular testing at community health centers in low-income countries should consider health worker perceptions about safety, identify plans for hazardous waste disposal, plan for power interruptions limiting charging of batteries, identify mechanisms to secure testing platforms and accessories, consider changes to streamline clinical and laboratory workflows to enable rapid testing and treatment initiation, and engage health center- and district-level leadership in monitoring performance and providing oversight.

## Background

TB remains the leading cause of death worldwide claiming an estimated 1.4 million people in 2019 [1]. Of the 10 million estimated annual cases, 3.6 million are unreported or undiagnosed and nearly 38% of those diagnosed are lost to follow-up prior to treatment initiation. Rapid and more sensitive diagnostic tests are critical to address these follow-up and treatment gaps. Xpert MTB/RIF (Xpert) is a semi-automated molecular test that simultaneously detects Mycobacterium tuberculosis complex (MTBC) and resistance to rifampicin (RIF) in less than 2 h with high sensitivity and specificity [2]. Xpert is now endorsed as a first-line test for TB by the World Health Organization and has been scaled up rapidly in most high burden countries [1]. However, Xpert scale-up has largely been limited to central health facilities due to power and other infrastructure requirements not available across all types of clinical sites where TB patients are evaluated.

Earlier studies of point-of-care Xpert testing reported faster availability of test results and more rapid treatment initiation for confirmed cases as a key benefit [3]. However, previous versions of the GeneXpert platform required a stable power supply and a temperature- and dust-controlled environment, which are lacking at many community health centers in high TB burden countries [4]. Newer Xpert testing platforms with integrated batteries and heat- and dust-resistant design are now available for deployment at community health centers. There are limited data on facility-and health worker-level factors that would promote successful adoption of such platforms for rapid TB testing and treatment initiation at community health centers.

Therefore, as part of a larger study whose objective was to conduct a cluster-randomized trial of a multi-faceted intervention based around onsite molecular testing for TB at community health centers in Uganda [5], we sought to understand health center readiness to implement onsite Xpert testing and identify strategies to enhance readiness that could be employed during intervention deployment.

## Methods

### Study setting and design

The parent trial on which this study is based was conducted in Uganda, which is one of 48 countries considered to have a high TB burden [1]. Earlier studies that investigated health system and contextual barriers to TB diagnosis in Uganda from a health workers perspective reported that challenges were experienced across the entire continuum of TB evaluation. Barriers reported include lack of training among health workers to confidently screen and diagnose TB, regular stock outs of drugs and other laboratory supplies required for TB diagnosis, low health worker motivation, and stigma against TB patients as the major barriers that required improvement [6, 7]. To understand the readiness for implementation before launching onsite Xpert testing, we conducted a qualitative study at participating community health centers. We selected six of the 20 health centers included in the trial to participate, aiming for diversity in terms of health center size and structure and urban vs. rural location. All six health centers were located in four districts in the central and eastern regions of Uganda. Three of the sites were Level III health centers providing mainly outpatient services including TB diagnosis and treatment. The other three sites were Level IV health centers that also had infrastructure enabling limited inpatient services and emergency surgeries. Four of the sites were in rural districts (sites 1, 3, 4, and 6) and one in urban (site 5) and semi-urban (site 2) districts. All sites were contacted prior to the assessment and agreed to participate.

Study design was informed by selected sub-domains of the Consolidated Framework for Implementation Research (CFIR). CFIR is a “determinant framework” describing constructs that are hypothesized to or have been demonstrated to influence implementation outcomes [8]. The framework’s developers identified areas of similarity and difference across existing frameworks and theories and consolidated them to create a framework that can be used to guide implementation research across a range of settings [9]. CFIR is a widely used implementation science framework for the purpose of evaluating readiness for implementation (i.e., the initial integration of interventions into settings) [10] and to optimize early and effective adoption [11], including in sub-Saharan Africa [12]. Similar to other studies that have employed CFIR for developing and analyzing qualitative data [13, 14], our study selected CFIR constructs based on our prior knowledge that the selected domains/subdomains (intervention characteristics, inner setting defined the complex features within which the implementation and interaction was done) were the most relevant to the study context. The constructs were used to develop the interview guide with relevant topical questions, organize, and describe the characteristics of the intervention to interviewees, capture perceptions from health workers regarding potential barriers or enablers to implementation, and develop themes for data analysis and interpretation [15] (Table 1). A recent systematic review [10] showed that CFIR-based assessments during the pre-implementation phase could help anticipate potential barriers before large-scale implementation in low resource settings.

**Table 1:** Key CFIR domains and constructs assessed

CFIR domain/constructs	Focus area assessed for Xpert implementation
I. **Intervention characteristics**	
a. Where the intervention originated (e.g., internal or external)	Perception of whether the intervention originated externally or as part of the National TB Program
b. Evidence strength and quality	Assessment of effectiveness of Xpert testing relative to the current TB diagnostic and treatment approach
c. Design quality and package	Perceptions on simplicity and quality compared to the standard test for TB (sputum smear microscopy)
d. Relative advantage	Perceived advantages of Xpert testing relative to smear microscopy for TB testing and diagnosis.
II. **Inner setting**	
a. Structural characteristics	Size of the health facility, number of health workers, and patient population
b. Individual stage of change	How site staff perceived their roles and how easily they could change their practices to accommodate onsite Xpert testing.
c. Compatibility	Assessment of how patient workload is handled and anticipated impact on workflow processes to facilitate same day testing and treatment with Xpert.
d. Networks and communication	Opinions on how information shared between individuals, across departments and in the whole health facility, including formal and informal communication and social networks (not specific to intervention). How Xpert will impact communication about TB diagnosis and treatment. Staff teamwork and ability to work together
e. Implementation/learning climate	
1) Tension for change	Degree to which staff perceive their current practices for TB assessment as needing change.
2) Relative priority	Shared opinions among health center staff about the importance of implementation of Xpert in the community health facilities.
3) Organizational incentives and rewards	Understanding the role of extrinsic incentives in the adoption of new practices and how they could best be deployed to promote implementation of Xpert.
4) Goals and Feedback	Assessment of the degree to which facility goals were communicated to staff, acted upon, and the extent to which performance evaluation from leaders aligned with goals.
III. **Characteristics of individuals**	
a. Knowledge and beliefs about the intervention	Staff receptivity to Xpert and attitudes and knowledge about key aspects of the intervention.

### Participants

Health workers involved in TB diagnosis and treatment at the 6 health centers were invited to participate in semi-structured interviews during planned site visits by members of the research team prior to implementation of the parent trial between February and April 2018. The goal of the site visits and interviews was to assess clinic work practices, staff experiences providing TB care, and readiness to adopt and implement onsite Xpert testing. Inclusion criteria were (a) age ≥18 years, (b) employed by the health center, and (c) involved in work related to diagnosis and management of TB. We used purposive sampling to select four to five health workers involved in TB evaluation at each health center. We aimed to capture diverse perspectives towards implementation by selecting health workers with a range of roles including medical officers (hold a Bachelor’s degree in Medicine and Surgery), nurses, laboratory technicians, and clinical officers (hold a Diploma in Clinical Medicine). We obtained written informed consent from all health workers who participated in the interviews. The study was approved by the Makerere University School of Public Health Higher Degrees Research and Ethics Committee and the University of California San Francisco Committee on Human Research. Participants received the equivalent of 3 USD as compensation for their time.

### Data collection methods

Two research staff who had prior experience with qualitative data collection (CO and TN) conducted interviews in English, lasting between 1 and 2 h. Interviews were audio-recorded. In addition, one or both research staff recorded detailed field notes of their observations of the physical layout of health centers including the size, distances between the blocks, and spaces available for health workers to examine patients. Other observations included interactions between health workers as they performed their roles, availability of electricity, and other ongoing activities at the health centers that might impact implementation. A debriefing meeting was held among research staff at the end of each day to review and consolidate the field notes. Interviews were professionally transcribed, and transcripts and hand written field notes were uploaded into Dedoose version 7.0.23, a web-based application for managing, analyzing, and presenting qualitative and mixed method research data [16].

### Data analysis

We used a combination of inductive and deductive content analysis to code and interpret interview transcripts and field notes. Deductive analysis included the use of CFIR-based constructs to develop categories that guided the structure of our code book. Transcripts were read by at least three members of the research team (TN, SA, MH, and PS), guided by the following steps: (1) codebook development including both a priori codes (Relative advantage, complexity, self-efficacy, leadership engagement, tensions for change, and knowledge and beliefs about the intervention) based on CFIR constructs and de novo codes (faster turnaround time for test results, reduced need to transport sputum samples to higher level health centers, concerns about security of GeneXpert devices and accessories, and safety risks for disposal of hazardous waste) based on reading transcripts and identifying key topics; (2) applying codes to transcripts, with at least two independent coders working on each transcript (TN, SA, MH, and PS); and (3) thematic development using coded data [17]. Themes were mapped to CFIR constructs, with additional categories created as needed for findings that did not fit within CFIR domains or constructs. All stages described above were collaborative and differences in interpretation were reconciled through discussion by research staff (TN, SA, MH, and PS). Field notes were also coded and analyzed in a similar manner to provide context for our interview findings.

## Results

Study findings were reported following the standards for reporting qualitative research (SRQR) guidelines [18]**.**

### Participant characteristics

All health workers invited for interviews agreed to participate. Health workers who took part in interviews included 6 nurses/nursing assistants, 6 clinicians, 6 laboratory directors/technicians, 1 medical officer, 2 health center directors, and 2 other staff involved in TB evaluation (Table 2).

**Table 2:** Health center and staff characteristics

Characteristics	Site 1	Site 2	Site 3	Site 4	Site 5	Site 6
Health center level	IV	III	IV	III	III	IV
Location	Urban	Peri-Urban	Rural	Rural	Urban	Rural
Population served	36,373	19,801	30,858	62,242	28,100	28,748
Role of participant	• Nurse • Clinician • Laboratory technician	• Nursing assistant • Clinician • Laboratory director • Community health worker	• Nurse • Clinician • Laboratory director • Community health worker	• Nurse • Clinician • Laboratory director • Health center director	• Nurse • Clinician • Laboratory technician • Health center director	• Nurse • Medical officer • Clinician • Laboratory director

Qualitative results are presented and organized by themes relevant to the three CFIR domains and constructs assessed, with key barriers and facilitators across domains summarized in (Table 3).

**Table 3:** Perceived barriers and facilitators for adoption of onsite Xpert testing

Barriers	Facilitators
▪ Unstable electricity ▪ Disposal of used cartridges ▪ Insufficient staff at health center to work on anticipated increased patient workload. ▪ Security and safety of GeneXpert devices in the laboratory. ▪ Fear to use the device without knowledge and training on use and maintenance. ▪ Anticipated increase in patient testing volume. ▪ Low staff interest in and attitude towards TB work. ▪ Need for increased supervision and performance monitoring.	▪ Overwhelming staff enthusiasm for onsite Xpert testing to improve patient care ▪ Diverse skill sets of health workers and teamwork ▪ Supportive and creative health facility leaders

### CFIR domain: intervention characteristics

#### Relative advantage

Health workers were all aware of the ongoing scale-up of Xpert testing in Uganda by the National TB Programme and commented that they had more confidence in Xpert results in comparison to the old technique, sputum smear microscopy, where TB could be missed. Xpert testing was also perceived to be simpler than smear microscopy: “*We shall like it because the method we are using isn’t so friendly*” *(Laboratory staff, site 3).* A lab staff at site 6 believed that same day testing would improve the patient-provider relationship by reducing wait times. She indicated that she and other lab staff often encounter patients who take out their frustration about delays on lab staff. Describing herself as “thick skinned,” she nonetheless said she would welcome rapid testing because it would result in fewer insults from patients. All health workers at all health centers believed onsite molecular testing could help overcome challenges including testing sputum samples quickly and providing results to patients in a timely manner: “We have been taking samples to Iganga [for Xpert testing] but l think once it is within our setting the patient turnaround time will be reduced...” (Laboratory staff, site 3). Many health workers referred to the GeneXpert device as something of great value to be treasured: “Actually we shall keep it like a golden glass” (Laboratory staff, site 5).

#### Complexity and adaptability of the intervention

Health workers at five sites, however, expressed concerns about the skills required to operate the GeneXpert device and how to troubleshoot anticipated problems with the devices: “Maybe if the machine is faulty and needs some repairs that might be normal. So, I don’t know whether you quickly come and pick it then you go and repair it from there or the repairing will be done from within” (Nurse, site 4). A lab assistant at site 1 emphasized the need for training to be able to use the GeneXpert device. At three sites, all lacking reinforcement in their laboratory windows, health workers noted potential security concerns that made them reluctant to store valuable equipment. “…the other thing is security... over the thieves. Because currently we are having more thieves” (Laboratory staff, site 5). Lab staff at site 2, which is located within the same compound as the district administration, were confident of the security system at the health center, noting that the site is well fenced off which would prevent burglary and trespassing. Recommendations suggested by the participants to address the anticipated security and safety issues included reinforced windows, door locking systems, and full-time security guards: “...there may be a need to beef up security in the lab by putting burglar proof in the windows because currently they are not locking” (Nurse, site 6). Health workers at more than half of the sites expressed concerns that power supply issues could impede implementation. Regular electricity interruptions, for example, last 2 days or more and could impact testing and results reporting. At two sites, the research team observed a clinic without electricity for a full day (12 h) that patients were requested to return to the health center for another visit to complete the TB diagnosis process. At another site electricity was unstable which interrupted the sputum testing process. Also, laboratory staff anticipated challenges with battery life and questioned the ability of batteries to support testing for an entire day in the event that power was not available for charging. “…our electricity system is not stable; we depend on electricity when it is there. When it is not there then everything is down...So, if we have a backup of power?” (Laboratory staff, site 6).

Additional concerns included safe disposal of used Xpert cartridges. At one site, a clinician also working as facility director expressed concern about the small size of the health center and lack of space to manage infectious waste: “does it [the GeneXpert system] have something like waste generated? How do we manage it?” (Clinician, site 5).

### CFIR domain: inner setting

#### Self-efficacy

Health workers at half of the sites expressed confidence that there would be the necessary flexibility among staff to act in multiple roles to get work done, viewing reducing TB burden in the community as an important motivator: “…motivation rate is high, case detection is high and if not in the entire region we are the people heading in identifying TB” (Laboratory staff, site 2).

A strong belief in teamwork was expressed by health workers at most sites “...people are motivated to do their work because there is teamwork and you know when there is teamwork, when you coordinate things will run smoothly. So that's the culture here…” (Nurse, site 6). However, health workers at two of the sites (sites 1 and 4) had apprehensions that availability of onsite Xpert testing would increase the number of patients tested and there may be insufficient capacity to handle the increased numbers: “It is a good program, only that I imagine a situation when you are on duty alone, you have to attend to those other patients…now, I don’t know how it will be whereby they will just be sent to the lab....” (Nurse, site 4). Similarly, a Medical Officer at site 6 felt that the one existing laboratory staff would be unable to meet the increase in patients referred for TB diagnostic services he anticipated once the intervention was implemented.

#### Feedback and communication

Health workers at most sites expressed a need for increased supervision during implementation to ensure progressive performance improvement: “You know most of these programs come but supervision during implementation sometimes is minimal. So, I hope this will be different...” (Medical Officer, site 6). Meetings were commonly used at all sites as platforms to disseminate information. Health workers further recommended that quality metrics reports could be discussed during regular meetings to promote quality improvement and performance tracking: “...Yes…like the monthly supervisions, if there is a comment that was made, we discuss that in our meeting. At such and such a time this was noted not done or done in a wrong way so, how do we improve? Who is responsible? Who can help us to be the overseer” (Nurse, site 1). However, the frequency of meetings varied across sites and was noted as a potential concern at some sites: “…it’s supposed to be monthly but I have seen some situations where we go for three months without sitting” (Clinical officer, Site 1). At half of the sites (sites 1, 3 and 4) health workers indicated that meetings facilitated information sharing which could overcome challenges ranging from staff performance, conflict resolution, and quality improvement plans. However, the other half (sites 2, 5 and 6) had irregular meeting schedules.

#### Leadership engagement

Health workers had divergent opinions about how leaders should be involved in interventions implemented at their sites. At Site 3, staff felt health center directors should be accessible to support programs introduced at the health center. However, participants felt ownership and accountability was lacking since anyone at the health center could be held responsible for new interventions that were introduced. A laboratory staff explained, “I would recommend the in charge of the health unit to get involved because the in charge is in better capacity to disseminate information to the rest of the staff…..”. In contrast, health workers at sites 4 and 1 applauded the oversight received from district health officers, as articulated by one clinician at Site 4: “As long as the DHO [ District Health Officer ] is aware, no one is going to sabotage any programme that is good and entering into the district. Because we serve one purpose and that is our client.” At the same time, leaders at two health centers (sites 4 and 5) empowered health workers to get involved in programs of interest to them and even encouraged them to take leadership roles.

#### Tension for change

Health workers at all sites acknowledged that onsite Xpert testing has the potential to solve most challenges experienced while using smear microscopy and the sputum referral system. Health workers at three sites (sites 1, 4, and 5) believed that onsite testing would reduce the transportation costs incurred to send sputum samples for testing at the centralized laboratories “...We shall minimize the transport costs we have been putting in for transporting those sputum samples, we shall be able to release results in time, and it will enable us to at least increase on the notification rate” (Nurse, site 5). Health workers at the same sites also noted that with same day testing, sites will no longer experience delays in patient results reporting as was the case with smear microscopy and sputum referrals. At one site, health workers anticipated that onsite Xpert testing would simplify work because it would be faster to process the tests while working on other laboratory related activities, which was not possible when using smear microscopy processes that required human involvement at every step of the process from the beginning to the end. “They [staff] will like it because it will have simplified the work. Instead of telling the patient that you go to Kayunga, they will be attended from here” (Nurse, site 4). Health workers at all sites identified inconsistent supply of laboratory commodities including sputum containers, test cartridges and TB drugs “... GeneXpert will be there but we won’t be doing anything. You can’t take a sample using your hand or a cup…” (Laboratory staff, site 1). One laboratory staff at site 2 described high patient satisfaction with the health center because patients perceive it [health center] as a district level health center. The participant believed onsite testing coupled with the convenient location of the health center would enhance patients’ experience further. “[Implementing the intervention] will meet my dream” (Laboratory staff, site 2).

#### Organizational incentives and rewards

When asked if recognition or other incentives would promote successful implementation, health workers at all sites described rewards and recognition as a promising means of gaining support and commitment from site staff to implement the intervention. Several types of incentives were recommended including paper certificates, money, T-shirts, and food for staff to share at the health center. The most popular across all sites was money. One site recommended hosting a meeting for staff from all sites to enable them to share experiences about the intervention and learn from each other. Despite the unanimous support for incentives, there was a lack of consensus across sites about how incentives should be distributed. For example, a staff at site 6 cautioned that incentives should be equitably distributed so as not to repeat the experience of earlier projects, in which incentives were applied to a selected group of staff resulting in resistance among those who did not benefit. Health workers at other sites shared the same opinion, as described by a lab staff: “I later realised that alone may not take me far and then the little money I could get I would call them and we share and tomorrow they do better work. They gained some motivation and that is the way of fighting attitude” (Laboratory staff, site 2). By contrast, a staff member at site 2 felt that only those staff involved with TB activities should be rewarded with a goal of attracting other health workers to pick up interest and participate in TB related work.

### CFIR domain: characteristics of individuals

#### Knowledge and beliefs about the intervention

All health workers believed the intervention would give them more confidence to improve work practices related to TB diagnosis and treatment. However, health workers at half of the sites also explained that they did not have sufficient knowledge to implement Xpert testing without additional training: “...there is a knowledge gap in some of the TB health workers in regards to TB...” (Laboratory staff, Site 1). A laboratory assistant at the same site felt that failure to provide training may affect her ability to use the test even when it would benefit patients. One laboratory director described how his personal experience working on TB related activities helped to change colleagues’ attitudes toward TB diagnosis and treatment “…I had to educate them about the importance…even me I had poor attitude and that help me to know the kind of people I am dealing with; I had experience. So, after that I brought them on board and started teaching them…” (Laboratory staff, site 2).

Health workers at all sites expressed a need for training in how the GeneXpert machine works, maintenance, and related processes pertaining to onsite Xpert testing. Health workers at site 4 proposed training schedules that did not overlap with clinical visits for patients. Such a schedule would provide health workers with sufficient time to participate in training while not compromising patient care. “Also, there has to be some training for the people who are going to be working on the machine since it is going to be a new machine brought to the lab” (Laboratory assistant, site 1).

Health workers believed GeneXpert training would create confidence among staff involved in Xpert testing by increasing their knowledge about TB diagnosis and treatment. “I think you people now that you have come you will also be carrying out some sort of education which will improve the knowledge of our staffs” (Medical officer, Site 6).

## Discussion

Our study to understand facility and health workers’ readiness to implement onsite molecular testing for TB revealed both similarities and differences across sites in implementation readiness and key barriers that needed to be overcome to facilitate effective uptake of onsite Xpert testing at community health centers in Uganda. The findings highlight the need for developing and testing co-interventions that target health system barriers when introducing novel diagnostic technologies for TB at community health centers in high TB burden countries.

Our study observed overwhelming interest and enthusiasm of health workers to participate in implementation of on-site Xpert testing. Health workers perceived Xpert testing as a superior technique relative to smear microscopy. They believed the new TB testing technique would make their work simpler and more efficient. In addition, support and endorsement from district and facility leaders gave health workers confidence in the new platform. An implementation study in Brazil [19] similarly showed that health center managers and health workers were excited following the introduction of onsite molecular testing. Many of the managers agreed onsite Xpert testing technology would increase health workers satisfaction with their work. However, these studies were conducted in health facilities located in cities and mostly interviewed health workers in senior positions. Our study involved Level III and IV health centers, which are the lowest level health centers that provide TB diagnostic and treatment services in Uganda. Interviews were conducted with various cadres of health workers involved in providing TB diagnostic and treatment services.

Although enthusiasm was high, we identified several key barriers to implementation of on-site Xpert testing. These included issues related to safety, security and maintenance of GeneXpert devices, concerns about infection risk and hazardous waste, electricity interruptions, inconsistent supply chain for laboratory commodities including test cartridges and sputum containers, inadequate clinical and laboratory workflows to handle anticipated increases in TB testing, and inadequate performance monitoring and oversight. Our results expand on findings from similar studies conducted in Mongolia and South Africa [20, 21] that reported similar barriers such as stock outs of laboratory supplies and inadequate trainings for health workers, and highlight specific issues that can be addressed when deploying point-of-care molecular testing platforms at community health centers.

Based on our findings, we modified our implementation strategy to involve district and health center leadership in planning and performance monitoring, thereby increasing stakeholder engagement. We involved district TB supervisors in health worker training to increase oversight and supervision, and modified training materials to highlight the benefits of onsite molecular testing to health workers (reduced workload and risk of infection) as was also done in India [22]. Beyond enhanced training, our findings highlight the need for developing and testing co-interventions such as workflow re-design performance feedback and performance-based incentives to address health system barriers to effective implementation of onsite molecular testing for TB.

Our study had some limitations. The assessment was done at only six health centers due to the short time period that was available to conduct this assessment before implementation of the cluster-randomized trial. However, we had balanced selection criteria across rural and urban health centers, level of health center and a diverse group of participants. The work was done in a single country, and additional barriers to implementation of onsite molecular testing for TB might be present at community health centers in other high TB burden countries.

## Conclusion

Our study generated a more nuanced understanding of modifiable contextual barriers and led to direct revisions of implementation strategies for the 20 participating sites. Findings also prompted the expansion of the implementation planning and oversight committee to include more health system stakeholders with the aim of improving trust and support for the program, and confirmed the need for health system co-interventions such as streamlining clinic workflows and performance feedback being evaluated in the parent trial. For future programs introducing innovative practices and devices in complex health care settings, pre-implementation assessment of individual perspectives, collaborative work processes, and institutional contexts is essential. Generating detailed empirical knowledge about the local readiness for implementation can lead to more effective tailoring of implementation strategies and may contribute to improved uptake and outcomes.

## Data Availability

All data generated and analyzed for this study are available with the corresponding author on request.
